# 

**Published:** 1894-01

**Authors:** 


### Communicated.

THE MISSOURI STATE DENTAL ASSOCIATION.—The twenty-ninth annual meeting of the Missouri State Dental Association was held at Excelsior Springs, Mo., July 11, 12, 13 and 14. The following officers were elected for the ensuing year:

President, W. E. Tucker, Springfield; 1st vice-president, J. S. Fry, Moberly; 2nd vice-president, Chas. L. Hungerford, Kansas City; Corresponding Secretary, Wm. Conrad, St. Louis; Recording Secretary, S. C. A. Rubey, Clinton; Treasurer, Jas. A. Price, Weston. Committee on Ethics, Frank Slater, Rich Hill; W. N. Morrison, St. Louis; C. L. Hungerford, Kansas City. Board of Censors, E. E. Shattuck, Kansas City; H. A. Cress, Warrensburg; E. B. Crane, California. Committee on Law, Jas. A. Price, Weston. Committee on New Appliances, John G. Harper, St. Louis.

The next meeting of this Association will be held at Excelsior Springs, Mo., on the first Tuesday after July 4th, 1894. WM. CONRAD, Cor. Sec'y.

---

RESOLUTIONS ADOPTED BY THE CHICAGO DENTAL SOCIETY ON THE DEATH OF DR. W. W. ALLPORT.—WHEREAS, In the death of Dr. W. W. Allport, a leader in our profession has fallen, and as a mark of our appreciation of his services and skill be it—

*Resolved*, That in his death the dental profession has lost a member whose extraordinary skill, as an operator, placed him among the foremost dentists of the world. His work in promoting the highest interests of the profession will ever be conspicuous, and the prosperity enjoyed by younger members is due in a great measure to his achievements.

*Resolved*, That a copy of this premable and resolutions be sent in proper form to the family of the deceased, and also to the dental journals for publication.

TRUMAN W. BROPHY,
A. W. HARLAN,
J. N. CROUSE,
Committee.

---

### Editorial, Etc.

### To Readers and Correspondents.

Communications intended for publication, and exchange journals and papers, should be addressed to the Editor of The American Journal of Dental Science, No. 845 N. Eutaw St. Baltimore, Md. All letters relating to the business of the Journal, or containing cash remittances, to be directed to Messrs. Snowden & Cowman, No. 9 W. Fayette Street, Baltimore, Md. Articles for publication should be received by the Editor at least two weeks previous to the first of the month on which the number in which it is designed for them to appear, is issued.

☞ The American Journal of Dental Science will contain fifty pages of reading matter in each number, making a volume of about Six Hundred pages,

EXCLUSIVE OF ADVERTISEMENTS

